# Transcriptome and metabolome analyses provide crucial insights into the adaptation of chieh-qua to *Fusarium oxysporum* infection

**DOI:** 10.3389/fpls.2024.1344155

**Published:** 2024-11-07

**Authors:** Yanchun Qiao, Jiazhu Peng, Bei Wu, Min Wang, Guoping He, Qingwu Peng, Yin Gao, Yuping Liu, Songguang Yang, Xiuchun Dai

**Affiliations:** ^1^ Vegetable Science Department, Guangzhou Academy of Agricultural and Rural Sciences, Guangzhou, China; ^2^ Vegetable Research Institute, Guangzhou Academy of Agricultural Sciences, Guangzhou, China; ^3^ Guangdong Key Laboratory for New Technology Research of Vegetables, Vegetable Research Institute, Guangdong Academy of Agricultural Sciences, Guangzhou, China; ^4^ South China Agricultural University, College of Horticulture, Guangzhou, China

**Keywords:** chieh-qua, *Fusarium oxysporum*, differentially expressed, metabolome, alternative splicing

## Abstract

**Introduction:**

Chieh-qua (*Benincasa hispida* Cogn. *var. Chieh-qua* How) is a wax gourd variety that is generally susceptible to infection and damage by *Fusarium oxysporum* during its cultivation. Therefore, analyzing the adaption mechanism of chieh-qua to *F. Oxysporum* infection is of great significance for cultivating resistant varieties.

**Methods:**

Through comparative transcriptome analysis, comparative metabolome analysis, integrated analysis of transcriptome and metabolome and between *F. Oxysporum* infected samples and control samples of susceptible lines

**Results:**

This study found that proteins such as NPR1, TGA and PR1 in plant hormone signal transduction pathway were up-regulated after infection, which may activate a series of plant secondary metabolic synthesis pathways. In addition, the expression of 27 genes in the flavonoid biosynthetic process in resistant lines after infection was significantly higher than that in susceptible lines, indicating that these genes may be involved in fungal resistance. This study also found that alternative splicing of genes may play an important role in responding to *F. Oxysporum* infection. For example, plant protein kinase genes such as EDR1, SRK2E and KIPK1 were not differentially expressed after *F. Oxysporum* infection, but the transcripts they produced differ at the transcription level. Finally, through comparative metabolome analysis, this study identified potentially functional substances such as oxalic acid that increased in content after *F. Oxysporum* infection. Through integrated analysis of transcriptome and metabolome, some differential expressed genes significantly related to differential metabolites were also identified.

**Discussion:**

This study provides a basis for understanding and utilizing chieh-qua’s infection mechanism of *F. Oxysporum* through analysis of the transcriptome and metabolome.

## 1 Introduction

Chieh-qua (*Benincasa hispida* Cogn. var. *Chieh-qua* How), a variety of wax gourd (*B. hispida*), is an important vegetable crop in the Cucurbitaceae family, which is widely distributed in South China and Southeast Asian countries (Xie and Peng, 2007; Xie et al., 2019). As a vigorous annual vine, chieh-qua immature fruits are consumed and are also known for being a rich source of essential nutrients, including proteins, vitamins, and minerals (Zaini et al., 2011; Liu et al., 2014). During growth, chieh-qua is subjected to a variety of environmental challenges, including biotic stresses (herbivores attacking and pathogen infection) and abiotic stresses (drought and high or low temperatures) (Verma et al., 2013). Among them, Fusarium wilt (FW) caused by *Fusarium oxysporum* (*F. oxysporum*) is one of the most serious soil-borne diseases, causing severe decreases in production and quality in chieh-qua (Xie and Peng, 2007).

*F. oxysporum* is a common soil fungus with broad pathogenicity across many plant species (Gordon and Martyn, 1997; Diez et al., 2014). The FW caused by this fungus inflicts severe damage to the agricultural production of various crops, including tomatoes, potatoes, cucurbits, maize, and sugarcane (Gullino et al., 2015). Globally, FW has become a serious plant disease, leading to significant losses in agricultural production and economic development. *F. oxysporum* spreads through soil and seed transmission, invading the roots and stems of plants, causing tissue necrosis and decay (Perkowski et al., 1997). In infected plants, *F. oxysporum* can enter plant tissues through root injuries or vascular systems, disrupting water and nutrient supply, resulting in symptoms such as wilting, withering, and death (Giachero et al., 2022).

To counter fungal infections, including *F. oxysporum*, plants have evolved a series of complex and sophisticated defense mechanisms, including resistance gene analogs (RGAs) and the mitogen-activated protein kinase (MAPK) signaling pathway (Sekhwal et al., 2015; Jiang et al., 2018). RGAs, as an important gene family in the plant genome, play a crucial role in the interaction between plants and pathogenic fungi. The proteins encoded by these genes typically possess specific domains related to the recognition and defense against pathogenic fungi, thus initiating appropriate defense responses to protect plants from pathogen invasion (Sekhwal et al., 2015). Meanwhile, the MAPK signaling pathway is considered a key regulatory factor in plant defense responses (Delplace et al., 2022). This signaling pathway can perceive pathogenic signals in the external environment and regulate plant resistance to pathogenic fungi by controlling gene expression and metabolic pathways (Meng and Zhang, 2013).

Transcriptomics and metabolomics are two important high-throughput sequencing technologies used to study changes in gene expression and metabolite composition in plants under different physiological conditions. In recent years, with the continuous development of sequencing technologies and the improvement of bioinformatics analysis methods, an increasing number of studies have shown that combined transcriptomic and metabolomic analysis is of significant importance in unraveling the complex regulatory networks in plant biological processes, addressing biological questions, and developing new varieties of crops. Transcriptomic–metabolomic analysis of grapes revealed novel information regarding the dynamics of grape ripening (Fortes et al., 2011). In a combined transcriptomic and metabolomic analysis of barley, *HvCERK1* was found to enhance barley resistance to *F. graminearum* (Karre et al., 2017). Candidate gene StWRKY1 identified through transcriptomic–metabolomic analysis regulates phenylpropanoid metabolites, enhancing potato resistance to late blight (Yogendra et al., 2015). Although the combined transcriptomic and metabolomic analysis technique is now well established, there is still a lack of such analysis for understanding the disease resistance mechanisms in the wax gourd.

In this study, two chieh-qua inbred lines (wilt-susceptible and wilt-resistant) were used for targeted metabolomic and transcriptomic comparisons after *F. oxysporum* infection. Both lines were derived from “feicui”, an inbred cultivar of chieh-qua common in Southern China, while the wilt-resistant lines were natural mutation isolated from “feicui”. Compared to the control, we identified a large number of DEGs and transcripts, some of which are RGAs, while others are involved in disease resistance-related pathways, including the MAPK pathway. Additionally, integrating the metabolome data, we found differentially expressed genes (DEGs) significantly associated with differentially expressed metabolites and constructed a network diagram illustrating the gene regulation of differentially expressed metabolites. These findings provide valuable resources for wax gourd defense against *F. oxysporum*.

## 2 Materials and methods

### 2.1 Plant material and growth conditions

Chieh-Qua (*B. hispida* Cogn. var. *Chieh-qua* How) inbred line “feicui” (FC), a common cultivar in Southern China, was provided by the Guangzhou Academy of Agricultural Sciences (Guangzhou Academy of Agricultural and Rural Sciences). The wilt-resistant lines were natural mutations isolated from “feicui” (**Supplementary Figure 1**). The seedlings were grown in 32-well plates filled with an aseptic organic substrate at 28°C/20°C day/night temperatures in a greenhouse under a 16-h light/8-h dark photoperiod for approximately 20 days until the second true leaf stage.

### 2.2 Inoculation with *F. oxysporum*

The *F. oxysporum* provided by the Plant Protection Research Institute Guangdong Academy of Agricultural Sciences was cultivated on PDA solid medium at 28°C in the dark for 4 days, then cultured in potato dextrose broth on a shaker at 180 rpm at 28°C for 3 days. The spore suspension was diluted to 1×10^5^ spores per milliliter with sterile distilled water.

Chieh-qua’s leaves from both wilt-resistant and wilt-susceptible lines isolated from FC were chosen as the subjects of this study (**Table 1**). Seedlings were infected with *F. oxysporum* by irrigation of the roots with a fungal spore suspension (3 mL per seedling) at the second true leaf stage. The true leaves of FC seedlings were harvested at 48 h after inoculation for RNA sequencing (RNA-seq) and metabolomic analysis. Three biological replicates were performed for each treatment, with 10 seedlings for each replicate, and each sample weighed approximately 3 g. Three sample groups were obtained: CK (control group, wilt-susceptible lines without pathogen inoculation), GB (wilt-susceptible lines with pathogen inoculation), and KB (wilt-resistant lines with pathogen inoculation). Two differential comparative analysis groups were established: wilt-susceptible (GB) vs. control (CK), as well as wilt-resistant (KB) vs. GB.

**Table 1:** Sample information.

Tissue site	Treatment	Name	Group name
Leaf	Control	T1	CK
Leaf	Control	T2	
Leaf	Control	T3	
Leaf	Resistance to wilt	F1	KB
Leaf	Resistance to wilt	F2	
Leaf	Resistance to wilt	F3	
Leaf	Susceptible to wilt	Q1	GB
Leaf	Susceptible to wilt	Q2	
Leaf	Susceptible to wilt	Q3	

### 2.3 RNA isolation and sequencing

The total RNA from wax gourd leaf samples subjected to different treatments was extracted using the CTAB (cetyltrimethylammonium bromide) method (Gasic et al., 2004), with the following steps: The leaf samples were ground into powder in liquid nitrogen and transferred to 2-mL centrifuge tubes containing 1 mL of preheated CTAB extraction buffer (the CTAB extraction buffer was preheated in a water bath at 65°C and supplemented with 2% mercaptoethanol). The mixture of liquid and powder was vortexed thoroughly and then incubated in a water bath at 65°C for 5 min before adding an equal volume of chloroform/isoamyl alcohol (volume ratio of 24:1). The mixture was centrifuged at 1,200 rpm for 30 min, and the supernatant was transferred to new 2-mL centrifuge tubes. The chloroform/isoamyl alcohol extraction and centrifugation steps were repeated once more, and the pellet was discarded. The supernatant was mixed with 4 mol/L LiAc and incubated at 4°C for 2 h before centrifugation at 1,200 rpm for 10 min. The supernatant was discarded, and the pellet was washed three times with 70% ethanol. After discarding the supernatant, the pellet was air-dried in a laminar flow hood. The RNA was dissolved in 30 μL of DEPC-treated double-distilled water after treatment with DNase I (Invitrogen) to remove genomic DNA contamination.

Equal amounts of RNA from each sample’s three biological replicates were used to construct cDNA libraries. RNA purity (OD_260/280_ and OD_260/230_) was measured using a NanoPhotometer spectrophotometer, and RNA concentration was accurately measured using a Qubit 2.0 fluorometer. RNA integrity was assessed using an Agilent 2100 Bioanalyzer. Subsequently, cDNA libraries were constructed, followed by sequencing using the Illumina HiSeq platform.

### 2.4 Identification of DEGs

The transcriptome sequencing data were initially processed using fastp (v0.19.5) (Chen, 2023) to remove low-quality sequences and adapters. Subsequently, HISAT2 (v2.2.1) (Kim et al., 2019) was employed to map the filtered reads (in fastq format) to the reference genome of wax gourd (Xie et al., 2019). The resulting BAM files were sorted using SAMtools (v1.18) (Danecek et al., 2021). Transcript assembly and quantification were performed using StringTie to assemble the mapped transcripts and generate a transcript annotation file (GTF format). STAR (v2.7.10b) (Dobin et al., 2013) was utilized to map the filtered fastq files to the reference genome with the newly assembled transcript GTF file. Finally, RSEM (Grabherr et al., 2011; Dobin et al., 2013) was used to quantify the transcripts. The transcript reads count was used to represent transcript resolution. The expression levels of genes were quantified from the transcriptome data mapped to the reference genome using featureCounts (v2.0.1) (Liao et al., 2014). Differential expression analysis of genes and transcripts with differential abundance between the resistance and susceptible lines relative to the control (KB vs. CK and GB vs. CK, respectively) was conducted using the R package DESeq2 (Wang et al., 2010). Transcripts and genes with |log2FoldChange| >1 and *p*_adj_ < 0.05 were considered differentially abundant or differentially expressed. The expression levels of genes and the abundance of transcripts were normalized using FPKM and presented accordingly.

### 2.5 Pathogenicity tests and fungal biomass evaluation

The open reading frame of *LOC120087936* and *LOC120075251* was amplified by PCR and inserted into the pBI121 vector. The primers used for plasmid construction are listed in **Supplementary Table 1**. The recombinant constructs, as well as empty plasmids, were transformed into *Agrobacterium tumefaciens* strain GV3101 using the freeze–thaw method, and then they were transiently expressed in *N. benthamiana* leaves through infiltration, as described previously (Ma et al., 2012).

Pathogenicity test assays were performed as previously described, with some modifications (Li et al., 2021). After 36 h of infiltration, the infiltrated plants were sprayed with fresh spore suspension (1 × 10^5^ conidia/mL) of *F. oxysporum* and transferred in a growth chamber under long-day conditions (LD, 25°C, 16 h light/8 h dark, light intensity of 150 μmol m^−2^ s^−1^). After a 1-week incubation, the plants were photographed using a digital camera. The experiment was repeated three times, and each treatment used six seedlings of *N. benthamiana*.

For the fungal biomass assay, a well-washed piece of infected *N. benthamiana* leaf (approximately 1 cm^2^) was used for DNA extraction using a Fungal DNA kit (Omega, United States) according to the manufacturer’s protocol. DNA-based qPCR was performed with 2× iTaq™ Universal SYBR Green Supermix (Bio-Rad, Hercules, CA, USA). Relative fungal biomass was calculated as a ratio (*FoEF1α*/*NtEF-1α*) represented by the equation 2 ^[CT(NtEF-1α)-CT(FoEF1α)]^ as previously described (Park et al., 2012). The primer pairs for qPCR are listed in **Supplementary Table 1**.

### 2.6 Gene functional enrichment analysis

The upregulated and downregulated DEGs selected from the two comparison groups (GB vs. CK and KB vs. GB) were subjected to Gene Ontology (GO) and Kyoto Encyclopedia of Genes and Genomes (KEGG) enrichment analysis, respectively, and the internal function enricher of the R language package cluster Profiler (Wu et al., 2021) was used to perform functional enrichment analysis (the threshold is *p* < 0.05, *q* < 0.05). Copy the output GO column and Qvalue column to Revigo (http://revigo.irb.hr/) (Supek et al., 2011) for processing and draw using CirGO software (https://github.com/IrinaVKuznetsova/CirGO) (Kuznetsova et al., 2019) GO enrichment analysis circle plot. KEGG enrichment analysis results were visualized with GraphPad.

### 2.7 RGA identification and construction of co-expression networks with transcription factors

The RGAugury pipeline (Li et al., 2016) was used to identify RGAs in the wax gourd genome, including four major categories: NBS, RLK, RLP, and TM-CC. Log2foldchanges of the differentially expressed RGA were used to draw the ridge plot using the R package ggridges and ggplot2 (Villanueva and Chen, 2019). The protein sequences of plant transcription factors (TFs) were downloaded from the plantTFDB4.0 database (Jin et al., 2017). Subsequently, the protein sequences of wax gourd were subjected to a comparative analysis against the downloaded plant TF protein sequences using the diamond blastp (Buchfink et al., 2021). Proteins from wax gourd exhibiting sequence similarity below this 1E-5 were considered as the TFs of wax gourd.

The Pearson correlation coefficient (PCC) between TFs and RGAs was calculated, and their significance was determined using the Benjamini–Hochberg (BH) method. TF–RGA gene pairs with a |PCC| > 0.8 and a *q*-value < 0.001 were considered co-expressed. The co-expression network was plotted using Cytoscape (Smoot et al., 2011).

### 2.8 Calculation of FST and pi values for RGAs in populations

The SNP data used in this study were sourced from Xie et al. (2019). To assess genetic differentiation among populations, the sliding window approach implemented in VCFtools (Danecek et al., 2011) was employed. Specifically, windows of 2,500 base pairs (bp) in size were moved along the RGA gene intervals and their 4,000-bp upstream and downstream regions with step sizes of 50 bp and 250 bp, respectively.

### 2.9 Metabolomic sample processing

The samples of both control and infected groups of wilt disease-resistant varieties were subjected to vacuum freeze-drying. Subsequently, they were ground into a powder using a grinding mill (MM400, Retsch) at a frequency of 30 Hz for 1.5 min within liquid nitrogen. Following this step, 100 mg of the powdered sample was dissolved in 1 mL of extraction solution (70% methanol solution). The dissolved samples were stored in a refrigerator at 4°C overnight, during which they were vortexed three times to enhance the extraction yield. Afterward, the samples were centrifuged at 10,000*g* for 10 min, and the supernatant was collected. The filtered samples were passed through a microporous filter membrane with a pore size of 0.22 μm and stored in sample vials for subsequent liquid chromatography–tandem mass spectrometry (LC-MS/MS) analysis.

### 2.10 Metabolome analysis and integration with transcriptome

Based on the Metware database (Metware Biotechnology Co., Ltd, Wuhan, Hubei, China) and publicly available metabolite information databases, substance qualitative analysis was conducted using secondary mass spectrometry data. During the analysis, isotope signals were excluded, as well as duplicate signals originating from K+ ions, Na+ ions, NH4+ ions, and fragments of larger molecules themselves (Chen et al., 2013). Metabolite quantification is accomplished through the use of a triple quadrupole mass spectrometer employing a multiple reaction monitoring (MRM) mode. After obtaining mass spectrometry data for different samples, peak areas of all metabolite spectra are integrated, and peak integration is corrected for the same metabolite across different samples (Fraga et al., 2010). Based on the results of partial least squares–discriminant analysis (PLS-DA), we can initially screen for metabolites that exhibit differences between different varieties or tissues. Additionally, we can further refine our selection by incorporating *p*-values or fold change values from univariate analysis. The combination of fold change and the variable importance in projection (VIP) from the OPLS-DA model is used to identify differential metabolites. The selection criteria are as follows: Metabolites with a fold change ≥2 or ≤0.5 are chosen. A fold change of 2 or more or 0.5 or less indicates significant differences between the control and experimental groups. Building upon the above criteria, metabolites with a VIP value ≥1 are selected. VIP values represent the strength of the impact of the intergroup differences for the corresponding metabolites in the model’s discriminative classification of group samples. Generally, metabolites with a VIP value of ≥1 are considered to be significantly different.

The PCC between the expression level of DEGs and the content of the differential metabolites was calculated, and their significance was determined using the BH method. DEGs and differential metabolites with a |PCC| > 0.8 and a *q*-value < 0.0005 were considered significant correlations.

## 3 Results

### 3.1 DEGs and functional enrichment

**Figure 1:**
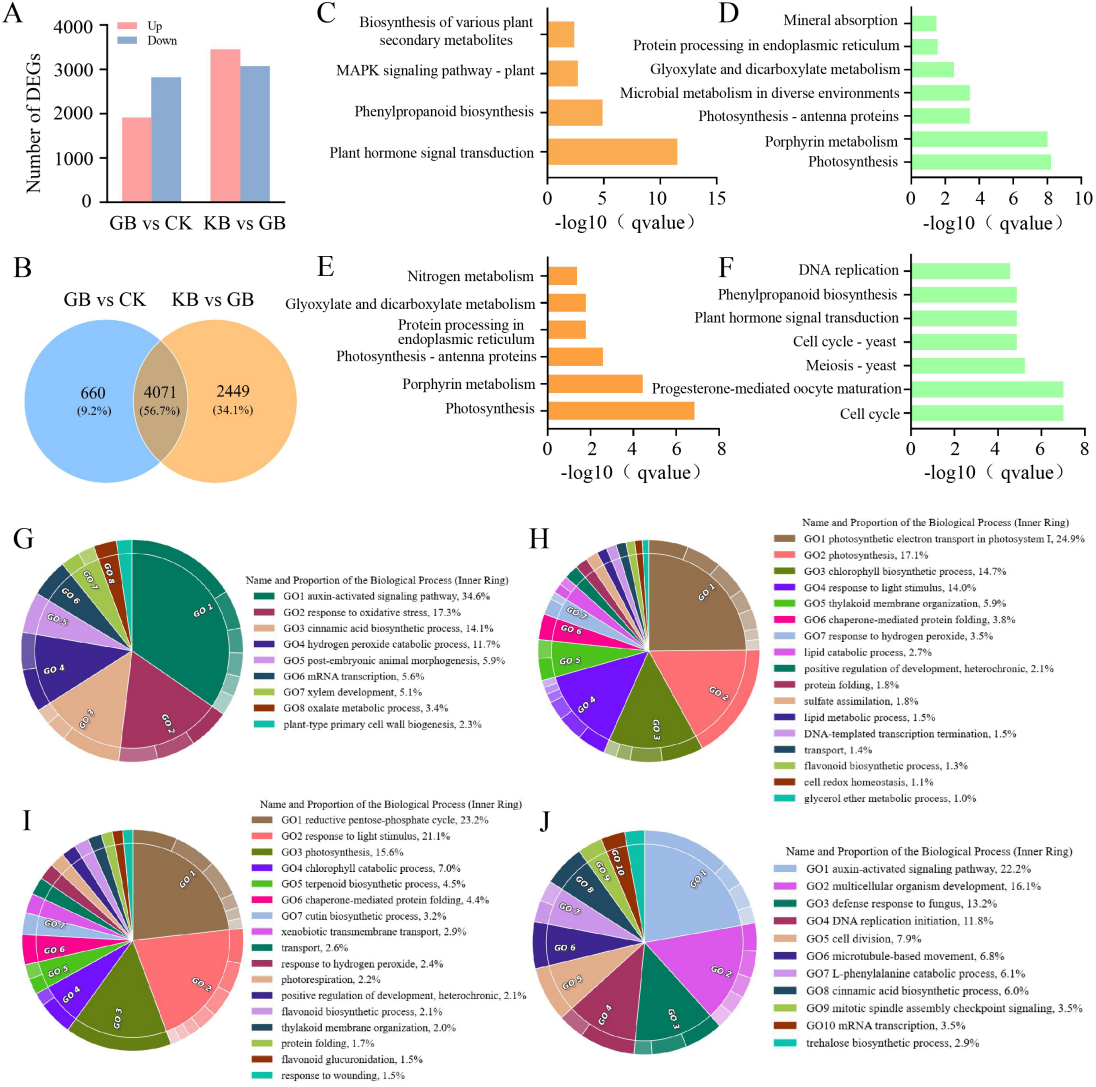
Gene differential expression analysis and functional enrichment analysis. **(A)** Bar chart depicting the number of differentially expressed genes between GB and CK, and between KB and GB. **(B)** Venn diagram illustrating the overlap of differentially expressed genes between GB and CK, and between KB and GB. Pathways significantly enriched with upregulated genes **(C)** and downregulated genes **(D)** in GB compared to CK, along with their significance values. Pathways significantly enriched with upregulated genes **(E)** and downregulated genes **(F)** in KB compared to GB, along with their significance value. Enrichment of upregulated genes **(G)** and downregulated genes **(H)** in GB compared to CK in biological processes under Gene Ontology (GO) terms. Enrichment of upregulated genes **(I)** and downregulated genes **(J)** in KB compared to GB in biological processes under GO terms.

*F. oxysporum* is a widely prevalent fungal pathogen in plants (Fravel et al., 2003), and the cultivation industry of chieh-qua is also affected by this disease. Therefore, understanding the corresponding mechanisms of chieh-qua against *F. oxysporum* infection is of significant value for controlling the pest and disease encountered during the chieh-qua cultivation process. In this study, RNA-seq was performed on samples from the CK, GB, and KB. Compared to CK, there were 1,912 upregulated DEGs and 2,818 downregulated DEGs in GB (**Figure 1A**). Compared to the GB inoculated with the *F. oxysporum*, 3,448 genes were highly expressed in KB, with 3,072 DEGs being downregulated. In the two comparison groups, there were 4,071 (56.7%) commonly shared DEGs, with 660 (9.2%) specific DEGs in GB vs. CK and 2,449 (34.1%) specific DEGs in KB vs. GB (**Figure 1B**). KEGG enrichment analysis of DEGs revealed enrichment of secondary metabolite biosynthesis pathways involved in biological defense functions among the upregulated genes after inoculation in the GB samples. (**Figure 1C**). At the same time, the rapid synthesis of secondary metabolites is activated by upstream signal regulation. The transport of plant hormones from their synthesis sites to target organs and their subsequent binding to receptors play crucial roles in plant physiology (Dermastia, 2019). The significantly upregulated genes were enriched in GO terms such as “hormone signal transduction” in the GB vs. CK groups, indicating that the infection process may activate multiple metabolite synthesis pathways. Simultaneously, many essential basic functions related to genes associated with processes such as photosynthesis appear to be downregulated (**Figure 1D**). Compared to GB, genes that were highly expressed in KB were enriched in pathways related to energy and growth, such as photosynthesis and nitrogen metabolism. This suggests that KB samples can maintain a better growth status after infection (**Figure 1E**). However, genes that were downregulated in KB were enriched in certain metabolic synthesis-related GO terms, such as phenylpropanoid biosynthesis (**Figure 1F**). This may be because these secondary metabolites do not participate in KB’s adaptation to F. oxysporum infection.

To explore the functions of DEGs between KB (wilt-resistant lines) and GB (wilt-susceptible), *LOC120087936* and *LOC120075251*, which exhibited high expression levels in KB, were selected for further analysis. Indeed, *LOC120087936* encodes a homolog of *Arabidopsis MILDEW RESISTANCE LOCUS O 13 (AtMLO13)*, which belongs to a large family of seven-transmembrane domain proteins that are specific to plants and are involved in conferring resistance to biotrophic powdery mildew fungus in barley. *LOC120075251* encodes a homologous version of *Arabidopsis* MDIS1-INTERACTING RECEPTOR LIKE KINASE2 (MIK2), which is a receptor heteromer involved in responding to various environmental stresses, including cell wall integrity sensing, salt stress tolerance, and resistance to *F. oxysporum* (Julkowska et al., 2016; Van der Does et al., 2017; Engelsdorf et al., 2018; Chaudhary et al., 2020). The plant transient expression vector pBI121 plasmid was used to create constructs of *35S: LOC120087936* and *35S: LOC120075251*. The *A. tumefaciens* harboring constructs were infiltrated into at least four leaves (per seedling) of *N. benthamiana* for transient expression. After 36 h of infiltration, two transient expressed lines were evaluated regarding their resistance to *F. oxysporum*. At 7 days after the wounded leaves were sprayed with *F. oxysporum*, the control tobacco leaves (CK, pBI121 plasmid only) were wilted and shorter. In contrast, the leaves of overexpression of *LOC120087936* and *LOC120075251* were growing well and appeared healthy (**Supplementary Figure 2A**). To further determine whether the expression of *LOC120087936* and *LOC120075251* affected the fungal growth in planta, we estimated the relative fungal biomass in the infected leaves by DNA-based quantitative PCR (q-PCR). The assays showed that the relative fungal biomass was lower in the expressed *LOC120087936* and *LOC120075251* plants compared with CK (**Supplementary Figure 2B**). These results showed that *LOC120087936* and *LOC120075251* play an essential role in the pathogenicity of *F. oxysporum*. The differential expression results in this study provide a resource for the functional validation of *F. oxysporum*-resistant genes.

GO enrichment analysis of upregulated and downregulated DEGs in GB vs. CK provided more comprehensive information for assessing gene functions related to *F. oxysporum* infection. Specifically, the upregulated DEGs in GB vs. CK were mainly enriched in pathways such as the auxin-activated signaling pathway, adaptation to oxidative stress, and cinnamic acid biosynthetic process (**Figure 1G**). These enriched secondary metabolite synthesis and regulatory pathways further underscore the important role of secondary metabolites in the adaptation to *F. oxysporum* infection. Similarly, the downregulated DEGs in GB vs. CK were mainly enriched in pathways such as sulfate assimilation, lipid metabolic process, glycerol ether metabolic process, response to light stimulus, and photosynthetic electron transport in photosystem I (**Figure 1H**), consistent with the KEGG enrichment results.

The upregulated DEGs in KB vs. CK were primarily enriched in pathways related to protein folding, transport, xenobiotic transmembrane transport, response to light stimulus, response to wounding, and flavonoid biosynthetic process (**Figure 1I**). Conversely, the downregulated DEGs in KB vs. CK were mainly enriched in pathways such as DNA replication initiation, L-phenylalanine catabolic process, multicellular organism development, auxin-activated signaling pathway, and defense response to fungus (**Figure 1J**). These findings provide further insights into the molecular mechanisms underlying the adaptation of KB to *F. oxysporum* infection, highlighting the complex interplay of various biological processes and pathways involved in the host–pathogen interaction.

### 3.2 DEGs involved in MAPK signaling pathways and salicylic acid signal transduction

**Figure 2:**
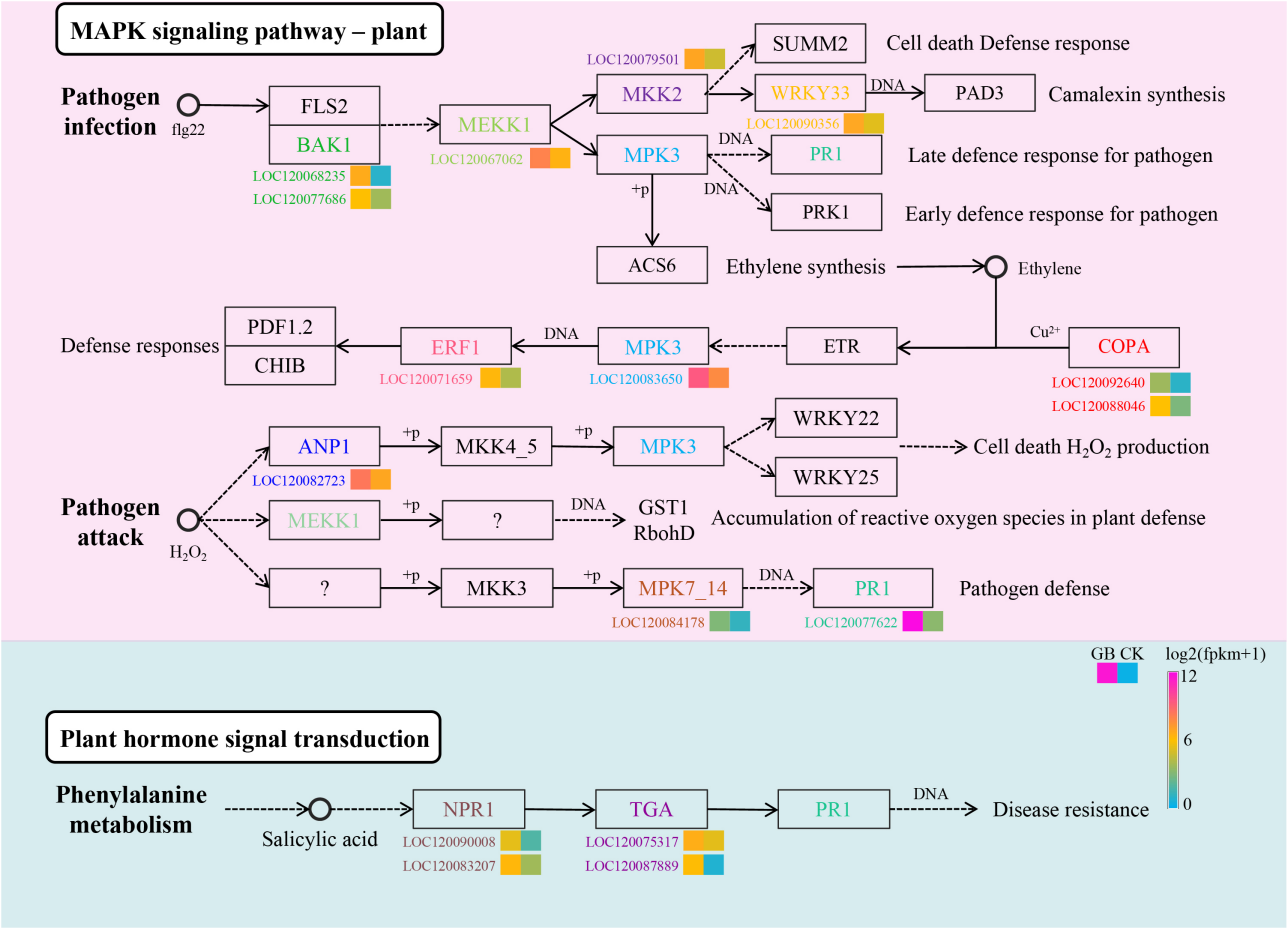
Identify the DEGs involved in the plant MAPK signaling pathway and plant salicylic acid signaling transduction.

Previous studies have demonstrated that the plant MAPK signaling pathway and plant hormone signaling transduction, including salicylic acid (SA), are key factors in regulating plant immunity. Here, we compared the gene expression changes of genes in the MAPK pathway in GB samples before and after infection (**Figure 2**). *BAK1* showed significantly upregulated expression after infection. Studies have shown that *BAK1* acts as a positive regulator in the MAPK signaling pathway (Chinchilla et al., 2007). Its upregulation activates downstream *MEKK1*, which is the starting point of several pathways formed by downstream MAPKs (Zipfel et al., 2004). Positive feedback responses activate various downstream pathways, such as the activation of camalexin synthesis through WRKY33, which is a secondary metabolite that inhibits bacterial and fungal infection (Koprivova et al., 2019). During the plant’s adaptation to microbial infection, various defense genes are regulated by ethylene (Ecker and Davis, 1987). The results of this study found that genes *COPA*, *MPK3*, and *ERF1* on this pathway showed upregulation after infection, revealing the role of the ethylene-regulated pathway in the adaptation to *F. oxysporum* infection. Similarly, genes *ANP1*, *MPK7/14*, and *PR1* involved in the activation of cell death, H_2_O_2_ production, and pathogen defense pathways were upregulated after *F. oxysporum* infection. SA is a plant hormone, and several genes related to its signal transduction, including *NPR1* (Nonexpresser of PR Genes 1), *TGA* (Transcription Factor GATA), and *PR1* (Pathogenesis-Related Gene 1), showed a similar expression pattern, significantly upregulated in GB compared to CK. These results not only suggest that the SA signaling pathway may play a role in the adaptation to *F. oxysporum* infection but also provide insights for further experiments to study its effects on downstream secondary metabolite synthesis.

### 3.3 Identification of RGAs and co-expression networks with TFs

**Figure 3:**
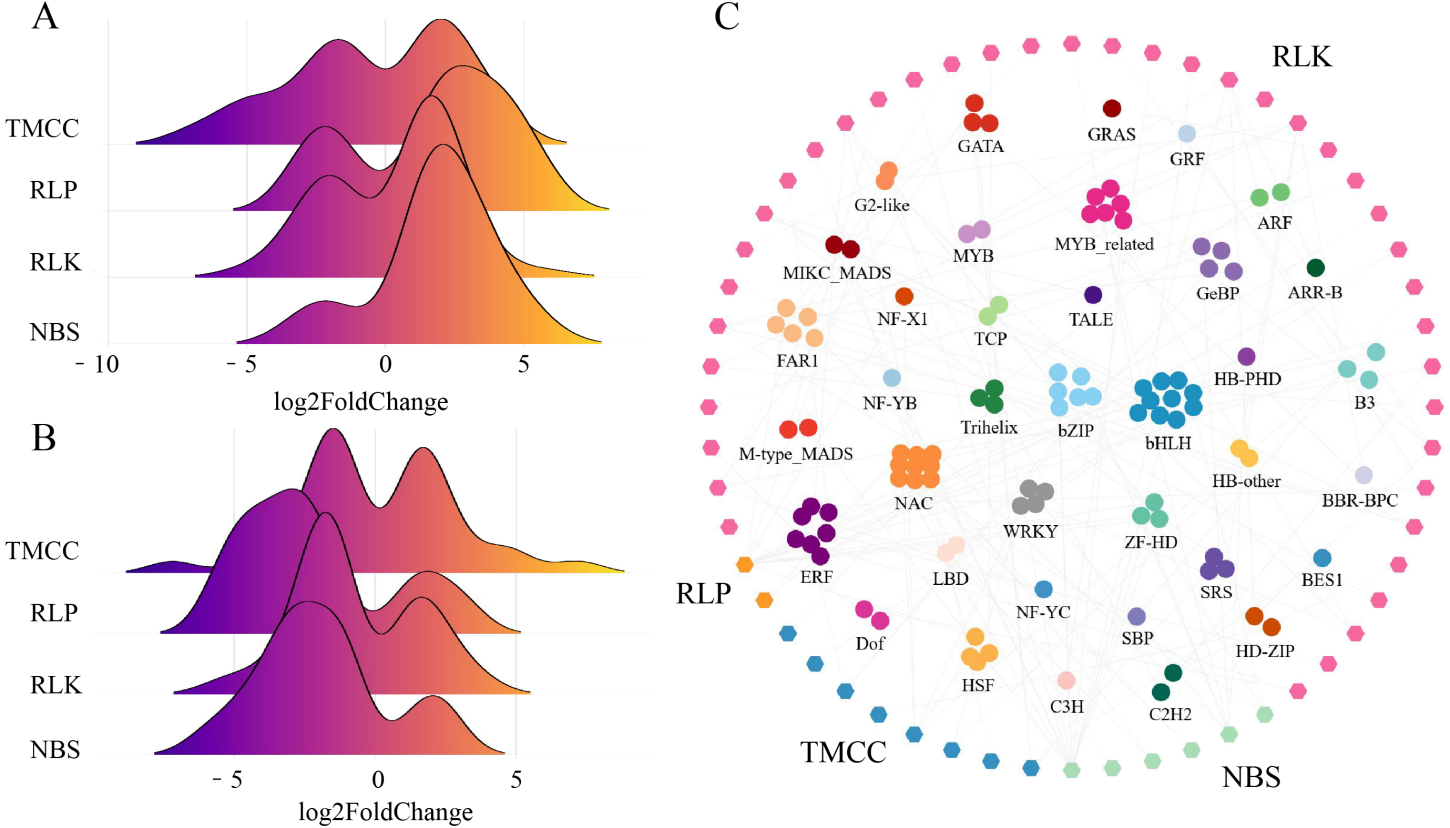
Expression and regulation of four differentially expressed RGAs (TMCC, RLP, RLK, and TM-CC). **(A)** Differential expression fold change stacked plot for RGA in GB vs. CK comparison analysis results. **(B)** Differential expression fold change stacked plot for RGA in KB vs. GB comparison analysis results. **(C)** The co-expressed network of TFs and differentially expressed RGAs.

RGAs are an important class of disease resistance genes. In this study, 664 RGAs were identified in the wax gourd, with 82, 36, 441, and 105 genes belonging to the NBS, RLP, RLK, and TM-CC gene families, respectively. As RGAs associated with plant resistance, differential expression analysis can identify candidate genes for further screening and application. From the distribution of RGAs’ log2FoldChanges in GB vs. CK (**Figure 3A**) and KB vs. GB (**Figure 3B**), it can be observed that some members of the TM-CC gene family not only respond to infection in GB but also show differences between GB vs. KB. A total of 23 TM-CC genes are differentially expressed in both GB vs. CK and GB vs. KB, indicating that these genes not only respond to *F. oxysporum* infection but also exhibit a stronger adaptation in resistant varieties. The number of differentially expressed RLP, TM-CC, and NBS in KB vs. GB is significantly fewer than that of the RLKs (177), with 17, 33, and 33 genes overlapping with those responding to *F. oxysporum* infection in GB, respectively.

To explore potential TFs regulating these RGAs, the PCC between TFs and differentially expressed RGAs was calculated. Using a threshold of |PCC| > 0.8 and *q*-value < 0.001, a total of 119 TFs were significantly correlated with RGAs (**Figure 3C**). Among them, the most abundant TFs were bHLH, which were mostly associated with the regulation of RLKs. There were 6, 2, 40, and 8 NBS, RLP, RLK, and TM-CC genes, respectively, potentially regulated by TFs. These results provide a data foundation for understanding the regulatory patterns of resistance genes in chieh-qua in the adaptation to *F. oxysporum* infection.

### 3.4 Genetic differences in RGAs among different populations

**Figure 4:**
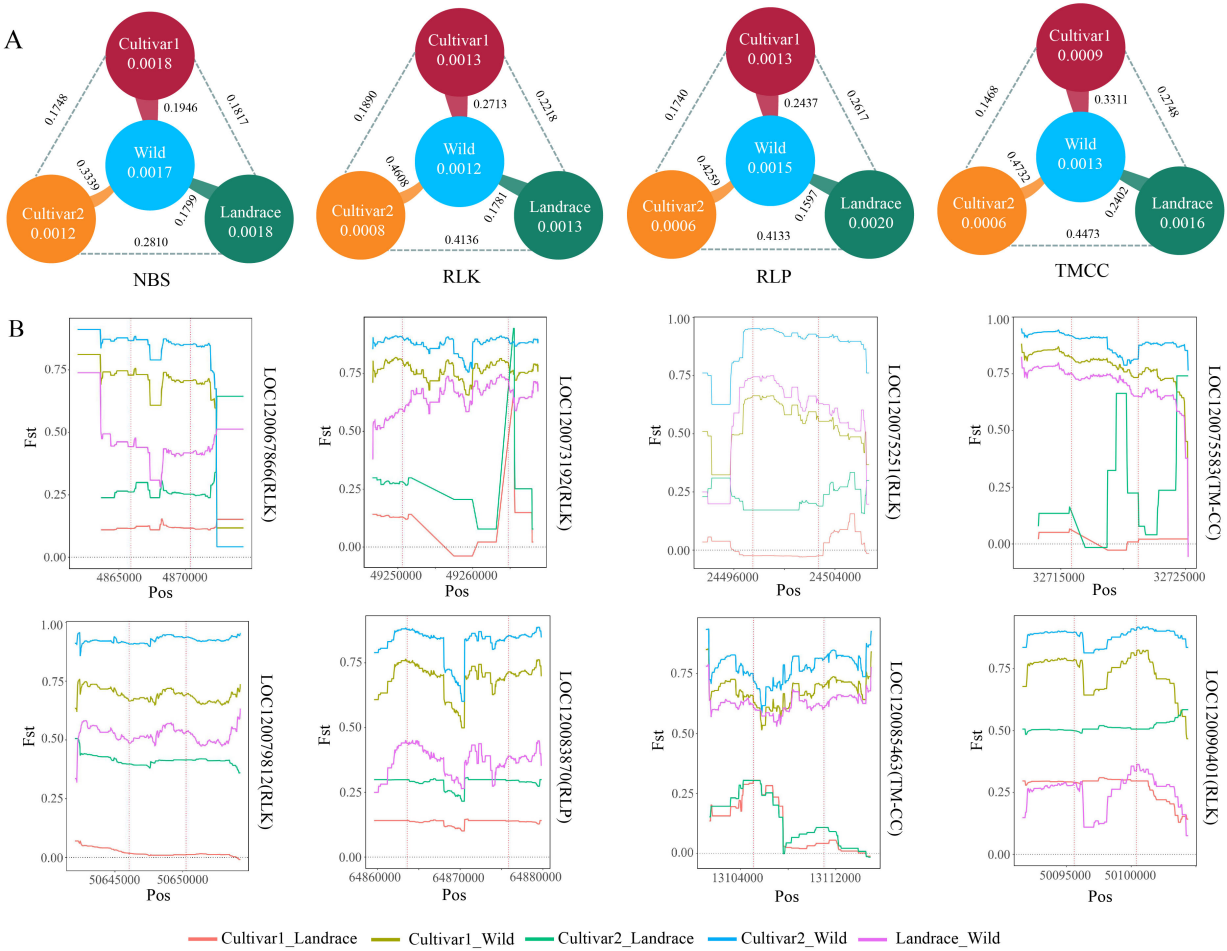
Population genetic analysis of RGA. **(A)** Differences in Fst values and pi values of differentially expressed NBS, RLK, RLP, and TMCC genes among Wild, Cultivar1, Cultivar2, and Landrace. The lines indicate the Fst values between two populations. The numbers inside the circles represent pi values of the populations. **(B)** Distribution of Fst values for differentially expressed RGA genes among different populations.

The study of Xie et al. divided the wax gourd population into cultivar1, cultivar2, landrace, and wild, as well as provided resequencing information (Xie et al., 2019), which laid the foundation for our analysis of selection signals for differentially expressed RGAs in the populations. The calculation of pi values for all RGAs showed that the diversity of RGAs in the landrace population was similar to that in the wild population (**Figure 4A**). However, the diversity of the TMCC, RLP, RLK, and TM-CC families in the cultivar2 population was the lowest, indicating a reduction in the genetic diversity of resistance genes in the cultivar2 population during breeding selection. Correspondingly, the Fst values between cultivar2 and wild were 0.3339, 0.4608, 0.4259, and 0.4732, respectively, indicating the possible presence of selection signals in the RGA regions between wild and cultivar2 populations.

To further screen for RGAs that may be under selection, this study analyzed the distribution of Fst values between populations for all differentially expressed RGA genes and their upstream and downstream 4-kb regions. Eight RGA genes (LOC12006786, LOC120073192, LOC120075251, LOC120075583, LOC120079812, LOC120083870, LOC120085463, and LOC120090401) had Fst values greater than 0.5 between cultivated (cultivar1 and cultivar2) and wild populations, indicating significant genetic differences between cultivated and wild populations within the 4-kb range upstream and downstream of these genes (**Figure 4B**). These results provided more candidate genes for resistance research.

### 3.5 Different expression patterns of the genes in the transcript levels

**Figure 5:**
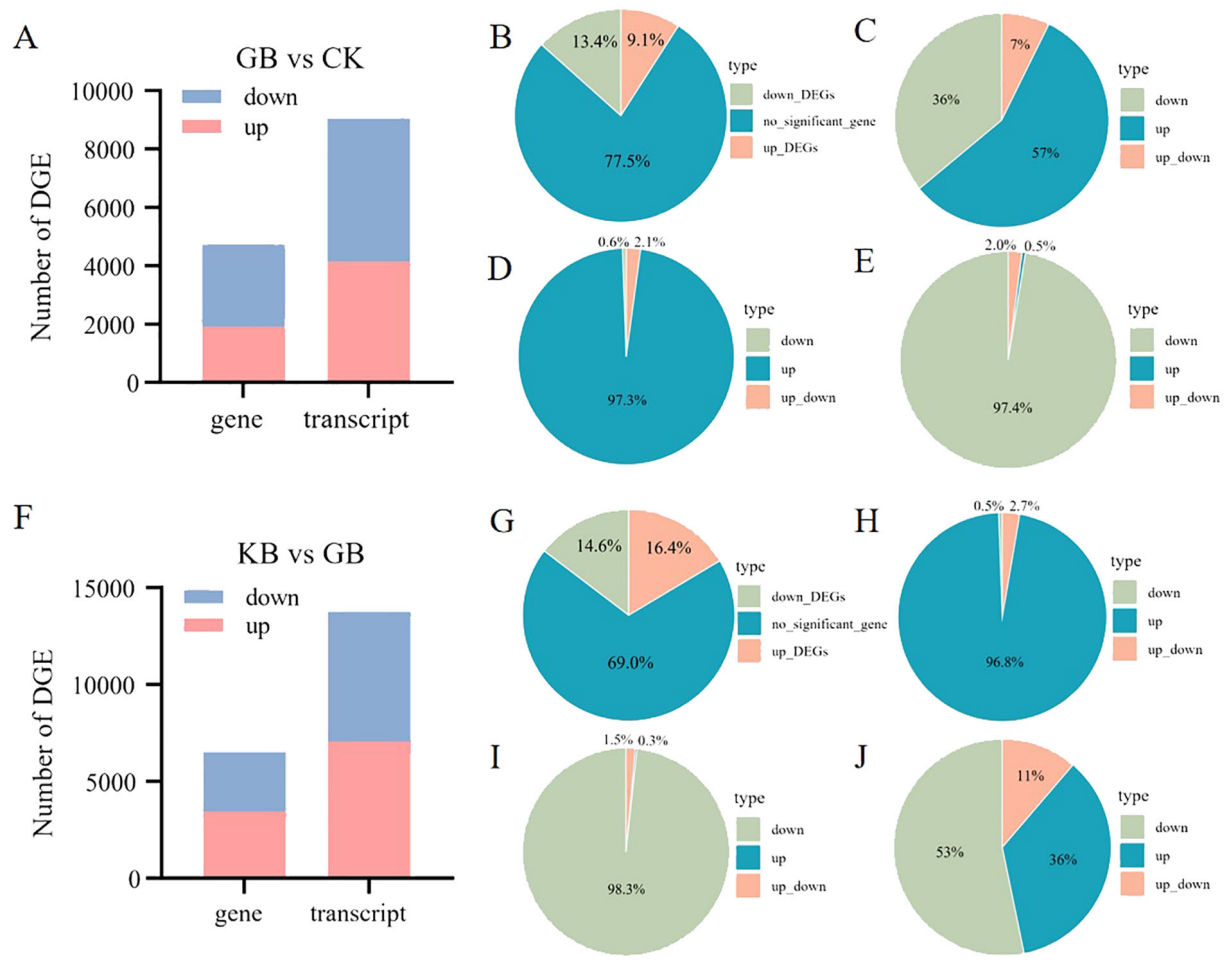
Differential gene and transcript resolution analysis in GB vs. CK and GB vs. KB. **(A)** Number of differentially expressed genes and transcripts between GB vs. CK. **(B)** Percentage of non-differentially expressed, upregulated, and downregulated genes in GB vs. CK. **(C–E)** Percentage of transcript differences expression pattern within non-differentially expressed **(C)**, upregulated **(D)**, and downregulated **(E)** genes in GB vs. CK. **(F)** Number of differentially expressed genes and transcripts between GB and KB. **(G)** Percentage of non-differentially expressed, upregulated, and downregulated genes in GB vs. KB. **(H–J)** Percentage of transcript differences expression pattern within upregulated **(H)**, downregulated **(I)**, and non-differentially expressed **(J)** genes in GB vs. KB.

Gene alternative splicing can lead to the existence of multiple transcriptional isoforms. In this study, a total of 71,771 transcripts were identified, originating from the expression of 21,004 genes. Comparing the differential expressed transcripts with that of genes can provide new insights into the role of gene alternative splicing in the adaptation of chieh-qua to *F. oxysporum* infection. Firstly, compared to CK, 1,912 upregulated and 2,818 downregulated DEGs were identified in GB, along with 4,158 upregulated and 4,891 downregulated differential transcripts (**Figure 5A**). The proportions of upregulated and downregulated DEGs, as well as non-DEGs in GB vs. CK were 13.4%, 9.1%, and 77.5%, respectively (**Figure 5B**). Among the 2,086 genes without differential expression but with differential transcripts, 152 genes had both upregulated and downregulated transcripts. Among the 16,274 genes with no differential expression, transcripts from 14,188 genes also showed no differential expression between GB and CK. Additionally, among the transcripts from the 2,086 genes, the percentages of transcripts with upregulation, downregulation, and both upregulation and downregulation between GB and CK were 57%, 36%, and 7%, respectively (**Figure 5C**). This indicates that different transcripts from the same gene may exhibit different expression patterns. Among the upregulated DEGs in GB vs. CK, 97.3% of genes also had upregulated transcripts (**Figure 5D**), while among the downregulated DEGs, 97.4% of genes also had downregulated transcripts (**Figure 5E**). Genes associated with functions such as Photosystem II and chloroplast envelope showed consistent expression patterns between transcripts and genes (**Supplementary Figure 3**). Comparatively, 3,448 upregulated and 3,072 downregulated DEGs were identified in KB compared to GB, along with 7,052 upregulated and 6,695 downregulated differential transcripts (**Figure 5F**). The proportions of upregulated DEGs, downregulated DEGs, and non-DEGs in GB vs. CK were 14.6%, 16.4%, and 69%, respectively (**Figure 5G**). The percentages of upregulated DEGs, downregulated DEGs, and genes with both upregulated and downregulated transcripts were similar to those in GB vs. CK (**Figures 5H, I**). However, downregulated transcripts enriched in pathways such as auxin polar transport and auxin homeostasis in upregulated genes deserve further attention (**Supplementary Figure 4**). Among the genes with no differential expression between KB and GB, there were 2,576 transcripts that were significantly differentially abundant, with proportions of upregulation, downregulation, and both upregulation and downregulation being 36%, 53%, and 11%, respectively (**Figure 5J**).

**Figure 6:**
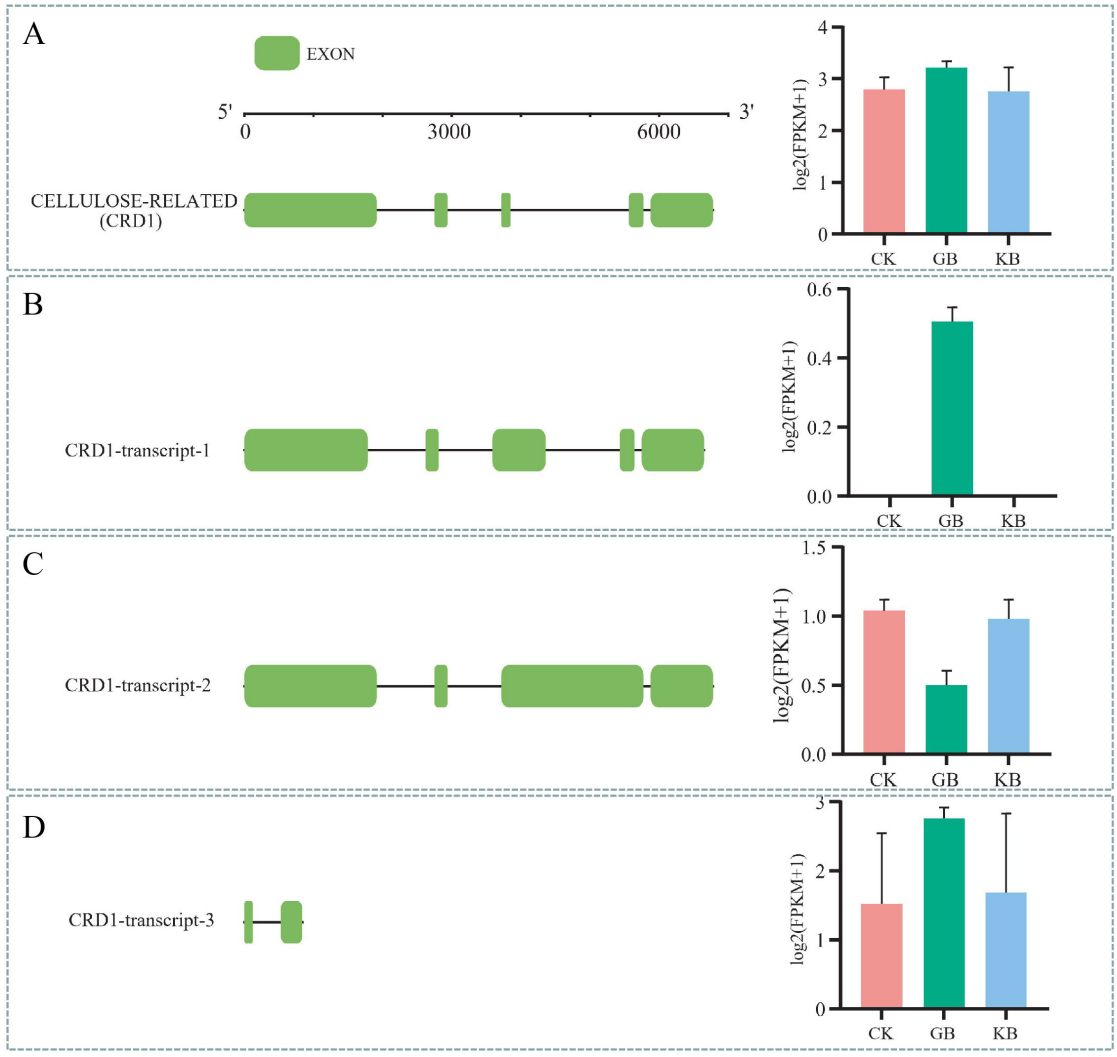
Diagram of alternative splicing for the LOC120069995. **(A)** The gene structure of LOC120069995 and bar graphs representing its expression values in different sample groups. **(B–D)** Exon structures of three transcripts generated by this gene and bar graphs representing their expression values in different sample groups.

LOC120069995 is a gene encoding a protein with the McbC_SagB-like_oxidoreductase functional domain. The gene structure and expression patterns of its transcripts indicate that LOC120069995 is not differentially expressed in both GB vs. CK and KB vs. GB (**Figure 6A**). The gene has five exons; its transcript MSTRG.25886.12 has five exons, with upregulated expression in GB vs. CK and downregulated expression in KB vs. GB (**Figure 6B**); MSTRG.25886.7 has four exons, with downregulated expression in GB vs. CK and upregulated expression in KB vs. GB (**Figure 6C**); MSTRG.25886.7 has two exons, with no differential expression in both GB vs. CK and KB vs. GB (**Figure 6D**). These results provided insights into the role of gene alternative splicing in the adaptation of chieh-qua to *F. oxysporum* infection.

### 3.6 Transcriptome and metabolome correlation network

**Figure 7:**
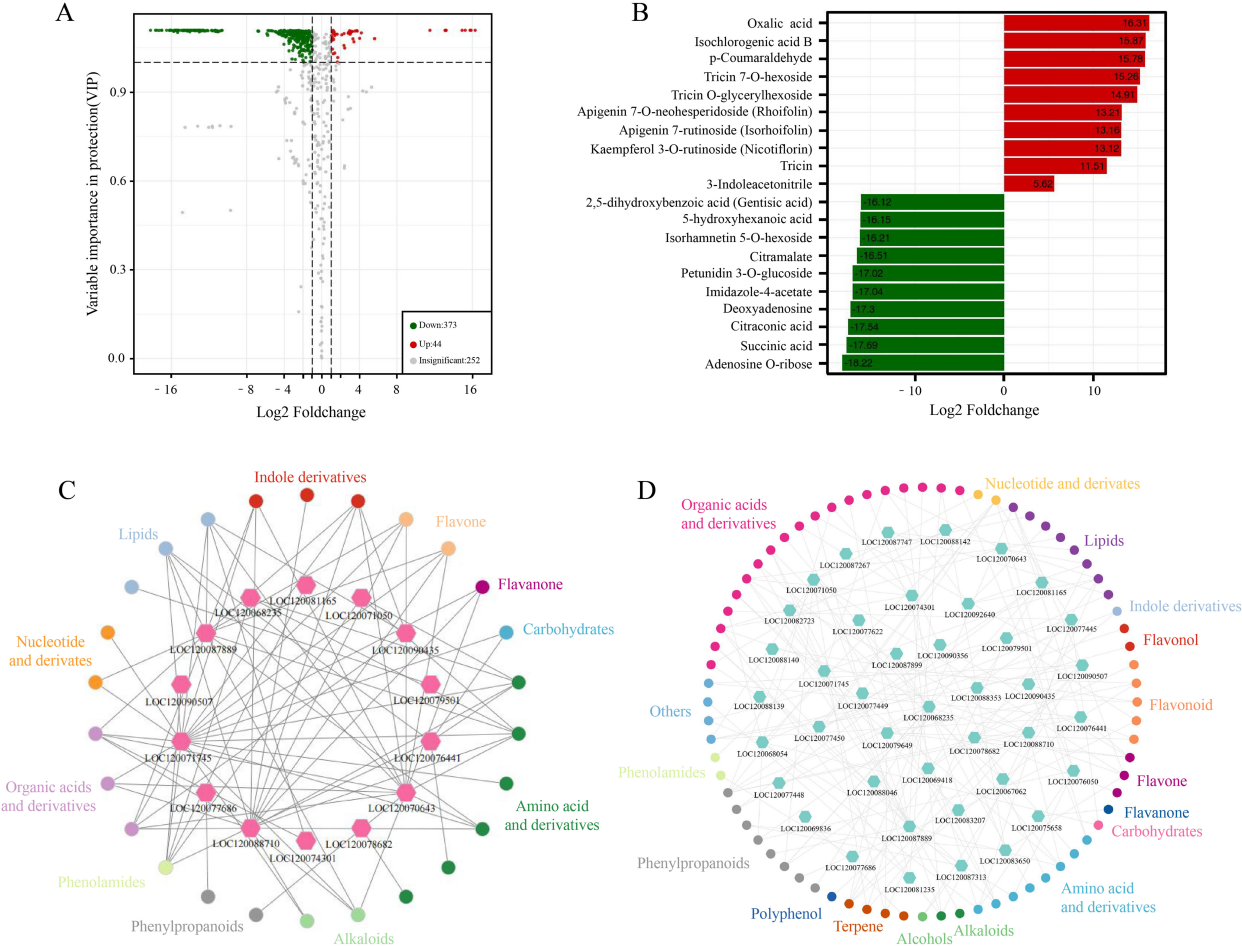
Metabolite analysis and correlation with transcriptomics. **(A)** Differential metabolite volcano plot in GB vs. CK: In this plot, each point represents a specific metabolite, with the *x*-axis indicating the logarithm of the quantitative fold change of a metabolite between two samples. Red points represent upregulated differentially metabolites, and gray points represent metabolites detected but not exhibiting significant differences in expression. **(B)** Metabolite differential fold change bar chart in GB vs. CK. **(C)** Network analysis of metabolites positively correlated with gene expression in GB vs. CK and KB vs. GB. **(D)** Network analysis of metabolites negatively correlated with gene expression in GB vs. CK and KB vs. GB.

In this study, metabolites from GB and CK samples were analyzed for metabolomics using ultra-performance liquid chromatography (UPLC) and MS/MS. Through annotation in the Metware database, a total of 672 known metabolites were detected (**Supplementary Tables 2**, **3**, **4**). Among them, 417 metabolites with annotation information showed differential contents between GB and CK, including 44 upregulated and 373 downregulated metabolites (**Figure 7A**). These metabolites belong to various categories such as flavanone, terpene, alkaloids, organic acids and derivatives, and phenylpropanoids. Metabolites of the organic acid and derivative types, such as oxalic acid and isochlorogenic acid B, exhibited the highest upregulation in content after *F. oxysporum* infection (**Figure 7B**). These results indicated the significant role of these substances in the adaptation of chieh-qua to *F. oxysporum* infection. Meanwhile, metabolites of the organic acid and derivative types, such as citraconic acid, showed a decrease in content after *F. oxysporum* infection, suggesting a potential inhibitory effect of *F. oxysporum* infection on their synthesis.

To further explore the relationship between genes and these metabolites, we performed a correlation analysis between DEGs involved in plant MAPK signaling pathway and plant SA signaling transduction identified in the GB vs. KB comparison group, and differential metabolites. The results revealed 14 DEGs and 26 metabolites (belonging to 11 classes) showing a positive correlation (**Figure 7C**). In particular, genes such as *LOC120071745* (Histidine-containing phosphotransfer protein 3), *LOC120077686* (BRASSINOSTEROID INSENSITIVE 1-associated receptor kinase 1), and *LOC120070643* (Respiratory burst oxidase homolog protein B) were associated with multiple metabolites in the positive correlation network. Lipids, flavanones, and other substances play important roles in the interaction between plants and pathogens (Aseel et al., 2019; Jeon et al., 2020). This study identified 647 genes, including *LOC120067029* (ATPase family AAA domain-containing protein 1-like), *LOC120068100* (zinc finger CCCH domain-containing protein 15-like), and *LOC120080874* (proteinase-activated receptor 2), whose expression was significantly positively correlated with these metabolites. Furthermore, 41 DEGs and 72 metabolites (belonging to 17 classes) showed a negative correlation (**Figure 7D**), among which organic acids and derivatives had 19 metabolites connected to other genes in the negative correlation network. Organic acids and derivatives may have more connections in the plant MAPK signaling pathway and plant SA signaling transduction process.

## 4 Discussion

*F. oxysporum* is a challenging fungal pathogen that spreads through water and soil. Therefore, uncovering the resistance mechanisms of the resistant line of chieh-qua against *F. oxysporum* through omics studies is of paramount importance for breeding resistant varieties. In this study, transcriptomic analysis revealed the involvement of a wide range of genes related to secondary metabolite synthesis in the adaptation to the infection. Genes in the SA signaling pathway, one of the plant hormones, were upregulated after *F. oxysporum* infection. This triggers plant immune functions, with one important pathway being the activation of genes related to secondary metabolite synthesis (van Butselaar and Van den Ackerveken, 2020). Plants have evolved complex and diverse biosynthetic pathways, particularly activating pathways for specific metabolites in the adaptation to biological stresses such as insects and fungi (Bai et al., 2023). Therefore, the analysis of genes involved in the production of responsive secondary metabolites is the basis for designing functional experiments. For example, in the flavonoid biosynthesis pathway, 27 genes including *LOC120078171* in KB were significantly expressed higher than in GB under *F. oxysporum* treatment, potentially enhancing the efficiency of flavonoid biosynthesis. Interestingly, in a cotton study, flavonoid biosynthesis was found to be associated with resistance to *F. oxysporum*, providing clues for exploring candidate resistance genes (Wang et al., 2022). In addition to secondary metabolites, the MAPK signaling pathway in plants is considered a crucial signaling pathway in plant defense (Thulasi Devendrakumar et al., 2018). This study found that they activate pathways such as Camalexin synthesis, which are associated with the synthesis of some metabolites related to plant defense (Nguyen et al., 2022).

During the lengthy process of evolution, plants have developed highly effective mechanisms to recognize and respond to pathogenic microbial invasions, with RGAs containing specific motifs and domains playing a crucial role (Sekhwal et al., 2015). In our study, we investigated the expression patterns of RGAs and found that TMCC responded differently to *F. oxysporum* infection compared to the RLK, RLP, and NBS families. Specifically, 33 members of the TMCC family showed significant upregulation in the KB vs. GB comparison. Additionally, considering population genomic information, the genetic diversity of TMCC in both cultivar1 and cultivar2 populations was significantly lower than that in the wild and landrace populations. To further explore RGAs, we conducted genetic differentiation analysis of the upstream and downstream 4-kb regions and gene regions of differentially expressed RGAs, identifying candidate genes such as *LOC120085463* and *LOC120075583* in the TMCC family. These genes exhibited significant genetic differentiation between cultivar1 and wild, between cultivar2 and wild, and between landrace and wild populations. Based on other large-scale population genomic studies, it is known that in the process of crop domestication, resistance tends to weaken, often requiring the introduction of wild genetic resources to enhance resistance (Guo et al., 2019; Zhao et al., 2019). Therefore, the identified RGAs in our study, which not only respond to *F. oxysporum* infection at the transcriptional level but also exhibit genetic differences between wild and domesticated populations, are important candidate genes for enhancing *F. oxysporum* resistance. Furthermore, we identified 119 TFs that may be involved in regulating differentially expressed RGAs, thereby expanding the range of candidate genes.

Because of the presence of alternative splicing, the abundance of transcripts often does not fully correlate with that of genes (Hu et al., 2022). Analyzing chieh-qua’s adaptation to *F. oxysporum* infection at the transcript level can provide new insights. Protein kinases are conserved regulatory factors in plants’ adaptation to pathogenic microbial invasion, catalyzing reversible protein phosphorylation reactions to regulate various cellular processes (Turrà et al., 2014). In this study, transcripts with a significant difference in abundance were identified within plant kinase genes that showed no differential expression at the gene level (**Supplementary Figure 3**), including TOR, ALE2, EDR1, SRK2E, and KIPK1 genes. This indicates that specific splicing patterns may play a role in the *F. oxysporum* infection process. Interestingly, this phenomenon has also been confirmed in other studies, such as the regulation of plant kinases SNC4 (SUPPRESSOR OF NPR1-1, CONSTITUTIVE4) and CERK1 (CHITIN ELICITOR RECEPTOR KINASE1) by gene splicing factors during plant immune adaptations. Therefore, based on differential transcript analysis, we can identify potential factors in chieh-qua’s adaptation to *F. oxysporum* infection, aiding in understanding the immune mechanisms of chieh-qua.

Secondary metabolites play a crucial role in the interactions between plants and other harmful organisms, making them a primary focus of chemical ecology research. This study employed comparative metabolomic analysis to elucidate the changes in metabolites during the *F. oxysporum* infection process in chieh-qua. Although the qualitative identification of unknown metabolites needs improvement, this study clarified the alterations in metabolites from existing libraries, laying the foundation for understanding the plant’s resistance at the metabolic level. For instance, oxalic acid, significantly elevated in GB, has been known to confer *Botrytis cinerea* resistance in tomatoes (Sun et al., 2019). The synthesis of functional secondary metabolites involves complex pathways, and identifying candidate genes in these pathways or genes regulating the pathways is crucial work. Transcription–metabolite integration analysis is a vital approach in this region (Schlüter et al., 2016). Through correlation analysis between transcriptome and metabolome, this study identified a series of genes potentially associated with important metabolite synthesis, providing a basis for further research into secondary metabolite synthesis. In summary, by comparing transcriptome and metabolome data, this study deciphered the changes in genes, transcripts, and metabolites in chieh-qua after *F. oxysporum* infection, offering insights into the responsive processes and laying an important groundwork for subsequent functional studies.

## Data Availability

The datasets presented in this study can be found in online repositories. The names of the repository/repositories and accession number(s) can be found in the article/**Supplementary Material**.

## Supplementary material

Supplementary Figure 1 Three treatments of wax gourd: CK (no treatment, left), GB (susceptible, treatment with *F. oxysporum*, middle) and KB (resistant, treatment with *F. oxysporum*, right).

Supplementary Figure 2 Pathogenicity assay of the transient expression of *LOC120087936* and *LOC120075251* in *N. benthamiana*. **(A)** Disease phenotype. The seedlings of tobacco at sixth or seventh leaf stage were selected for infiltration. Before and after 1-week incubation with spores of *F. oxysporum*, the seedlings were photographed, respectively. Scale bar = 5 cm. **(B)** Relative biomass assay. The relative *F. oxysporum* growth was measured by [2 ^CT (NtEF-1α)-CT (FoEF1α)^ ×100] using q-PCR. Values are means ± SE based on three independent experiments. Means with different letters are significantly different from each other (one-way ANOVA, *p* ≤ 0.05). “ND” means not detected.

Supplementary Figure 3 GO enrichment analysis column chart of three types of differentially expressed genes (upregulated, downregulated, and no significance). Red represents genes with differentially expressed transcripts that are only downregulated, green represents genes with differentially expressed transcripts that are only upregulated, and yellow represents genes with differentially expressed transcripts that are both downregulated and upregulated.

Supplementary Figure 4 KEGG enrichment analysis column chart of three types of differentially expressed genes (upregulated, downregulated, and no significance). Red represents genes with differentially expressed transcripts that are only downregulated, green represents genes with differentially expressed transcripts that are only upregulated, and yellow represents genes with differentially expressed transcripts that are both downregulated and upregulated.

Supplementary Table 2 The quantitative results of sample metabolites obtained in this study.

Supplementary Table 3 Differential metabolites in GB_vs_CK.

Supplementary Table 4 Differential metabolites in KB_vs_GB.
