# Supplementary material for: Transcriptome and metabolome analyses provide crucial insights into the adaptation of chieh-qua to Fusarium oxysporum infection
Source: Front Plant Sci. 2024 Nov 7;15:1344155. doi: 10.3389/fpls.2024.1344155 (PMC11578706; doi:10.3389/fpls.2024.1344155)
Supplement: Supplementary file 1 [file Table1.docx]

Table S1. Primers used in this study

| **Primer name** | **Primer Sequence (5'-3')** | **Reference** |
| --- | --- | --- |
| **Construction of transient expression vector** | | |
| LOC120087936-Up | CCCTCGAGgtcgacTAGTatggatataatttctccggtc | Present work |
| LOC120087936-Down | TCTCCTTTGCTAGTCATttcctcaacatatgaagaag | Present work |
| LOC120075251-Up | CCCTCGAGgtcgacTAGTatggctactcaccgccaacaac | Present work |
| LOC120075251-Down | TCTCCTTTGCTAGTCATattatttgcatcttccatttcaag | Present work |
| DNA-based qPCR analysis of fungal biomass | | |
| q*FoEF1α-*F | GCTGGTGACTCCAAGAACGA | Liu et al., 2019 |
| q*FoEF1α-*R | CATCTTGACGATGGCGGAGT | Liu et al., 2019 |
| qNtEF-1α-F | TGAGATGCACCACGAAGCTC | Schmidt et al., 2010 |
| qNtEF-1α-R | CCAACATTGTCACCAGGAAGTG | Schmidt et al., 2010 |

Liu, S., Wu, B., Yang, J., Bi, F., Dong, T., Yang, Q., et al. (2019). A cerato-platanin family protein FocCP1 is essential for the penetration and virulence of *Fusarium oxysporum* f. sp. *cubense* tropical race 4. *Int. J. Mol. Sci.* 20(15):3785. doi: 10.3390/ijms20153785.

Schmidt, G.W., Delaney, S.K. (2010). Stable internal reference genes for normalization of real-time RT-PCR in tobacco (Nicotiana tabacum) during development and abiotic stress. Mol Genet Genomics 283, 233–241. https://doi.org/10.1007/s00438-010-0511-1.
