# Supplementary figures and images for: Transcriptome and metabolome analyses provide crucial insights into the adaptation of chieh-qua to Fusarium oxysporum infection
Source: Front Plant Sci. 2024 Nov 7;15:1344155. doi: 10.3389/fpls.2024.1344155 (PMC11578706; doi:10.3389/fpls.2024.1344155)

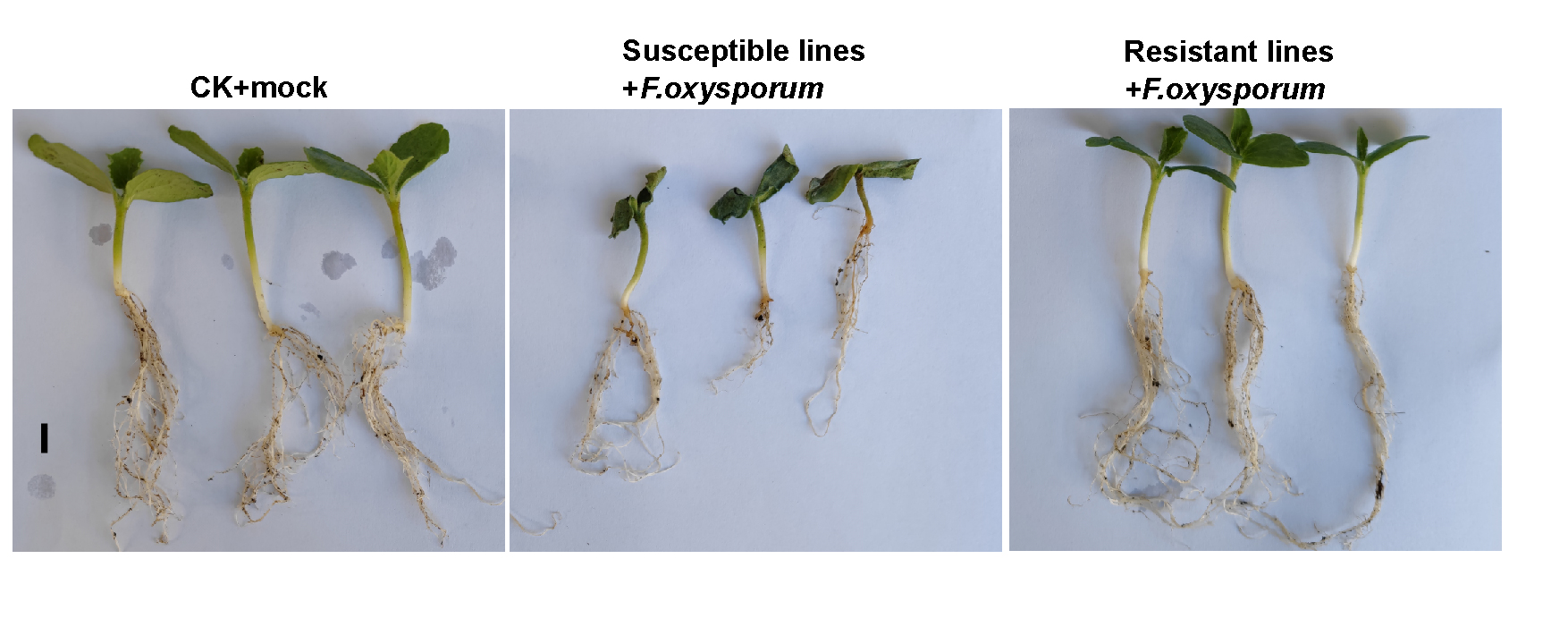

Supplement: Supplementary Figure 1 — Three treatments of wax gourd: CK (no treatment, left), GB (susceptible, treatment with F. oxysporum, middle) and KB (resistant, treatment with F. oxysporum, right). [file Image1.jpeg]

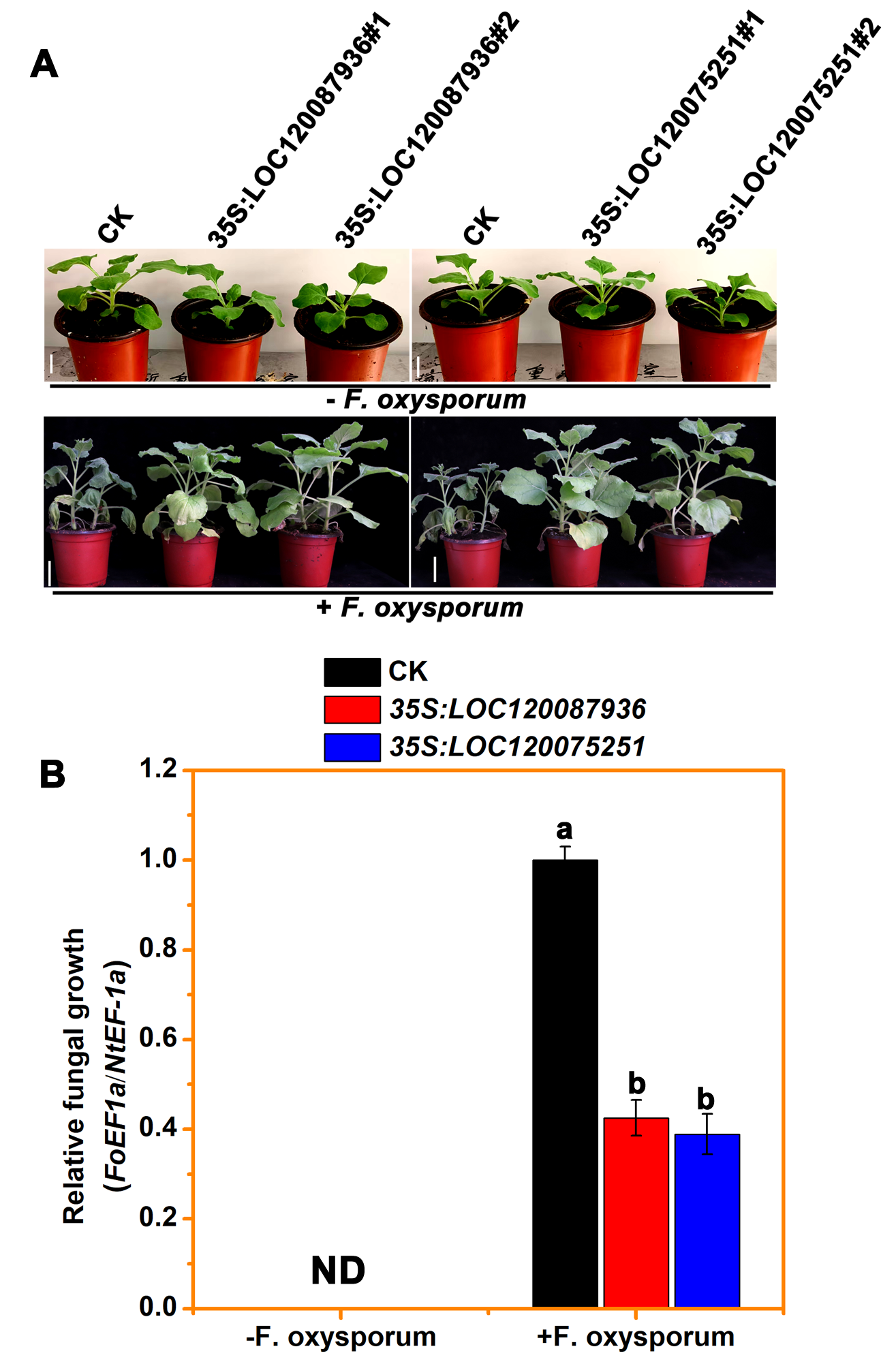

Supplement: Supplementary Figure 2 — Pathogenicity assay of the transient expression of LOC120087936 and LOC120075251 in N. benthamiana. (A) Disease phenotype. The seedlings of tobacco at sixth or seventh leaf stage were selected for infiltration. Before and after 1-week incubation with spores of F. oxysporum, the seedlings were photographed, respectively. Scale bar = 5 cm. (B) Relative biomass assay. The relative F. oxysporum growth was measured by [2 CT (NtEF-1α)-CT (FoEF1α) ×100] using q-PCR. Values are means ± SE based on three independent experiments. Means with different letters are significantly different from each other (one-way ANOVA, p ≤ 0.05). “ND” means not detected. [file Image2.tif]

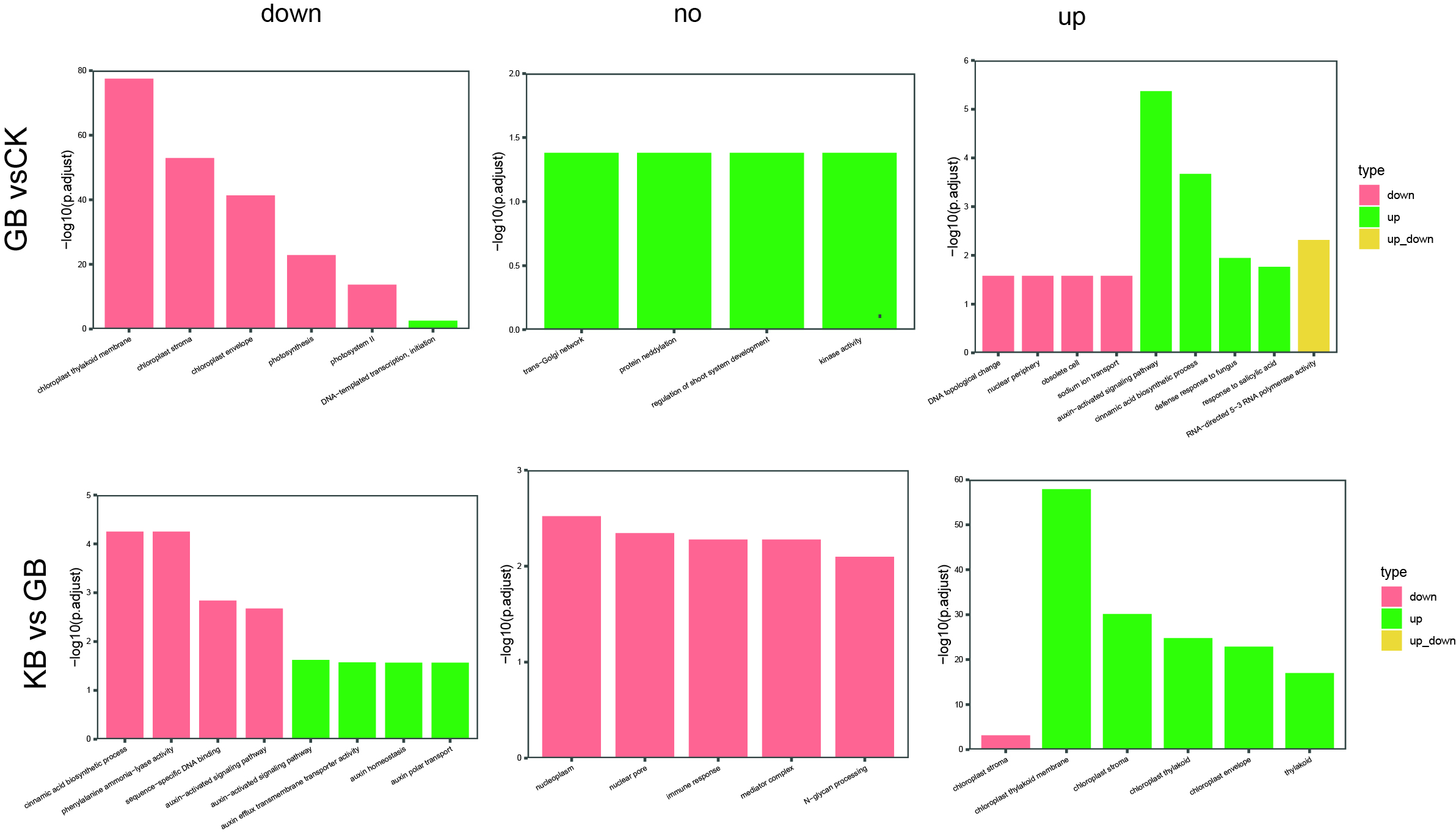

Supplement: Supplementary Figure 3 — GO enrichment analysis column chart of three types of differentially expressed genes (upregulated, downregulated, and no significance). Red represents genes with differentially expressed transcripts that are only downregulated, green represents genes with differentially expressed transcripts that are only upregulated, and yellow represents genes with differentially expressed transcripts that are both downregulated and upregulated. [file Image3.jpeg]

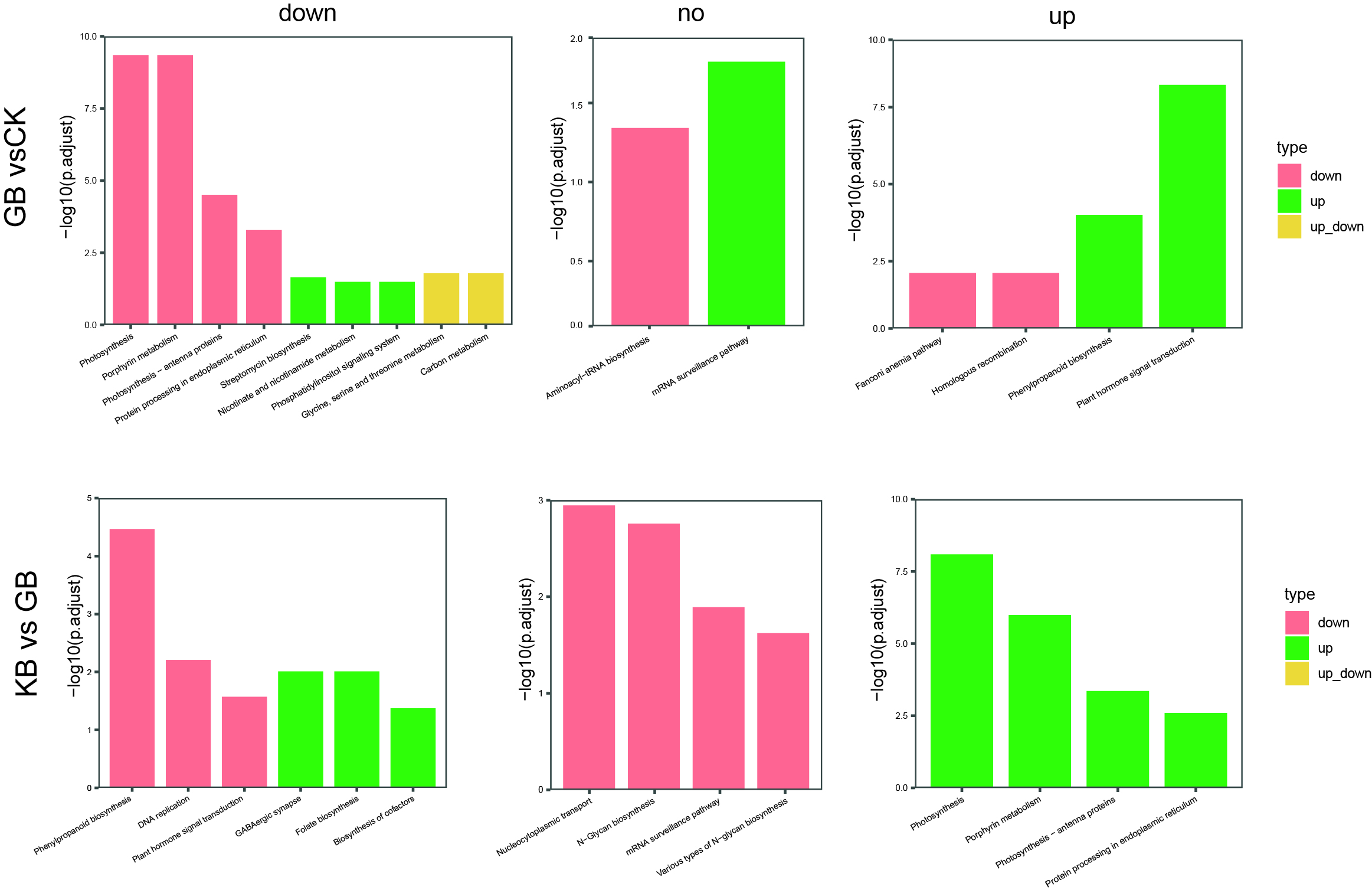

Supplement: Supplementary Figure 4 — KEGG enrichment analysis column chart of three types of differentially expressed genes (upregulated, downregulated, and no significance). Red represents genes with differentially expressed transcripts that are only downregulated, green represents genes with differentially expressed transcripts that are only upregulated, and yellow represents genes with differentially expressed transcripts that are both downregulated and upregulated. [file Image4.jpeg]
